# Climate change and epilepsy: Time to take action

**DOI:** 10.1002/epi4.12359

**Published:** 2019-09-20

**Authors:** Sanjay M. Sisodiya, Hayley J. Fowler, Iain Lake, Rosemary O. Nanji, Kinga Gawel, Camila V. Esguerra, Charles Newton, Aideen Foley

**Affiliations:** ^1^ Department of Clinical and Experimental Epilepsy UCL Queen Square Institute of Neurology London UK; ^2^ Chalfont Centre for Epilepsy Chalfont St. Peter UK; ^3^ School of Civil Engineering and Geosciences Newcastle University Newcastle upon Tyne UK; ^4^ School of Environmental Sciences University of East Anglia Norwich UK; ^5^ Centre for Molecular Medicine Norway Faculty of Medicine and Department of Pharmacy Faculty of Mathematics and Natural Sciences University of Oslo Oslo Norway; ^6^ Department of Experimental and Clinical Pharmacology Medical University of Lublin Lublin Poland; ^7^ Medical Sciences Division University of Oxford Oxford UK; ^8^ Department of Geography Birkbeck College University of London London UK

**Keywords:** antiepileptic drug, biodiversity, genetic, global warming, infection, seizures

## Abstract

Climate change is the biggest challenge facing humanity today. The associated global warming and humidification, increases in the severity and frequency of extreme climate events, extension of the ranges of vector‐borne diseases, and the consequent social and economic stresses and disruption will have major negative consequences on many aspects of health care. People whose resilience to change is already impaired may suffer disproportionately from these environmental changes, which are of unprecedented reach and magnitude. There has been little connection made so far between climate change and epilepsy. We briefly review the history of climate change science and the subsequent response of the global scientific community. We consider how climate change effects might in general affect health and disease. We consider some of the underlying complex interactions that, for example, favor the spread of vector‐borne diseases and how climate models operate and may help plan for global and local changes. We then speculate specifically on how these generic ideas may apply specifically to epilepsy. We consider these impacts at levels from molecular to the epidemiological. Data are sparse, and there is undoubtedly a need for more information to enable better estimation of possible effects of climate change on care in epilepsy. We also consider how the professional activities of those involved in epilepsy health care might contribute to global carbon emissions, for example, through flying for conference attendance. Healthcare organizations across the world are already considering, and responding to, many of these issues. We argue for more research in this area, but also for action today. Actions today are likely to generate cobenefits for health care, including care in epilepsy, resulting from efforts to decarbonize, mitigate effects of climate change that has already happened, and plan for adaptation to climate change.

**Key Points**

- Climate change is already affecting many aspects of daily life and health care, and has the potential to overwhelm healthcare systems.
- Warming and humidity changes will strongly affect human disease risks, such as ranges of vector‐borne infections, stress, and biodiversity loss.
- Many of the accompanying environmental, infrastructural, and socioeconomic changes will adversely impact the epilepsies at several levels.
- For the sake of people with epilepsy, professionals should consider their own professional contributions to climate change.
- More research is needed; professionals can already take action.

## 1 INTRODUCTION

Climate change, dominated by climate warming, is accepted by the majority of informed authorities as a clear feature of the Anthropocene era, and is becoming the lived experience shared by many of us globally, as we comment with increasing frequency about the unseasonal weather and the environment transforming around us. The effects of climate change will be pervasive, with no sphere of human endeavor likely to be untouched. As the 5th assessment report from the Intergovernmental Panel on Climate Change (IPCC) states: “Continued emission of greenhouse gases will cause further warming and long‐lasting changes in all components of the climate system, increasing the likelihood of severe, pervasive and irreversible impacts for people and ecosystems. Limiting climate change would require substantial and sustained reductions in greenhouse gas emissions which, together with adaptation, can limit climate change risks.”^1^

The risks to human health posed by climate change have already received extensive attention. The fourth edition, published in 2018, of the Lancet Countdown on health and climate change stated climate change was “the biggest global health threat of the 21st century,” warning that failure to take action could lead to disasters that “disrupt core public health infrastructure and overwhelm health services.”^2^ A further Lancet commission report details the interaction between significant healthcare challenges (a “syndemic”), working as multipliers that exacerbate unitary global challenges, such as obesity, undernutrition, and climate change.^3^

Several authorities and organizations have launched programs around the effects of climate change. The Global Climate and Health Alliance, formed in 2011, “works to tackle climate change and to protect and promote public health” and provides useful links and resources (http://climateandhealthalliance.org/). Several international groups, nations, and regions have their own healthcare initiatives focused on climate change, such as the Medical Society Consortium on Climate and Health in the United States (https://medsocietiesforclimatehealth.org/), the UK Health Alliance on Climate Change (http://www.ukhealthalliance.org/), Doctors for the Environment Australia (https://www.dea.org.au/), the Regional Institute of Health Medicine and Research (Rajasthan, India) (http://www.rihmr.org/), the European Environment and Health Youth Coalition (http://www.eehyc.org/), and the UK NHS (https://www.sduhealth.org.uk/policy-strategy/reporting/nhs-carbon-footprint.aspx), carbon emissions from which are estimated to account for between 4% and 6% of the total UK carbon footprint. The World Health Organization has made clear the health benefits of mitigating climate change far outweigh the costs.^4^ The US healthcare provider Kaiser Permanente announced in 2018 that it would become carbon neutral by 2020.^5^

Funders are increasingly supporting research into the health effects of climate change, typically with a focus on mitigation strategies. It seems unlikely that any human disease area will escape the pervasive anticipated and unforeseen consequences of climate change. Noting that the epilepsies are strongly influenced by environmental factors, we wished to start to consider how climate change might affect the epilepsies, to raise awareness within the epilepsy community of these issues, and to facilitate and promote efforts by people affected by epilepsy, families and carers, and epilepsy professionals, to begin to address what action we can take. Individuals may already be taking steps in their own private lives, for example, by working out their own carbon footprint (https://footprint.wwf.org.uk/#/ ‐ a very easy to use and enlightening tool) and taking effective actions.^6^ Patients, clinicians, and scientists across the world, as a community, may have ideas and initiatives for shared observations and mitigation measures to reduce the potential added burden of climate change for those with epilepsy. We here consider the history of climate change, review the current state of science and global efforts to tackle climate change, describe how climate change can affect human health and disease, and then address the particular impacts there may be on the epilepsies, how health care in the epilepsies might contribute to climate change, what can be done to mitigate the effects, and how progress might be achieved.

## 2 CLIMATE CHANGE: A BRIEF SURVEY OF THE HISTORY AND CURRENT STATUS

Climate change science has a much longer history than most people realize, dating back to the 19th century when Joseph Fourier recognized that the Earth's atmosphere kept the planet warmer than would be the case in a vacuum. He effectively discovered the greenhouse effect: the passage of visible light waves through the atmosphere to the earth's surface, their absorption, and re‐emission as infrared radiation, which can then be absorbed by certain types of gases in the atmosphere, increasing surface temperatures. Eunice Newton Foote was the first to suggest that the warming effect of the sun would be increased in the presence of carbon dioxide (CO_2_: then called “carbonic acid”). Her work was presented by Professor Joseph Henry at the American Association for the Advancement of Science meeting in August 1856. John Tyndall then examined the absorption of infrared radiation in different gases, discovering the greenhouse gases (GHG) CO_2_, methane, and water vapor in 1859. Swedish scientist Svante Arrhenius was the first to link human activity to global warming. In 1896, he published “On the Influence of Carbonic Acid in the Air upon the Temperature of the Earth,” calculating that a doubling of atmospheric CO_2_ (now called the “*climate sensitivity”*) would give a total warming of 5‐6°C. In later work, he revised this downward to 4°C.^7^ This is not dissimilar to the range reported by the IPCC’s 5th Assessment Report from the latest generation of global climate models.

Events then jump almost 100 years to the modern era when the first conference on climate change, a joint United Nations Environment Programme (UNEP)/World Meteorological Organization (WMO)/International Council for Science (ICSU) Conference on the "Assessment of the Role of Carbon Dioxide and Other Greenhouse Gases in Climate Variations and Associated Impacts," in 1985 concluded that GHG "are expected" to cause significant warming in the next century. This led to the establishment of the Intergovernmental Panel on Climate Change (IPCC) in 1988, whose role is to “assess the scientific, technical and socio‐economic information relevant for the understanding of the risk of human‐induced climate change,” and the United Nations Framework Convention on Climate Change (UNFCCC), an international environmental treaty adopted on May 9, 1992. The IPCC has issued a series of Assessment Reports once every 5‐6 years—published in 1990 (First Assessment Report: FAR), 1995 (Second Assessment Report: SAR), 2001 (Third Assessment Report: TAR), 2007 (Fourth Assessment Report: AR4), and 2013/2014 (Fifth Assessment Report: AR5)—and supplemental and special topic reports that describe the state of scientific understanding at the time each report is prepared. Although there have now been five Assessment Reports (and the 6th is under construction), the range of potential warming with a doubling of atmospheric CO_2_ has not changed substantially from the estimate made with quite simple climate models in the FAR, or indeed by Arrhenius in 1896, with both the FAR and AR5 suggesting a range from 1.5 to 4.5°C.

Alongside this, the UNFCCC objective is to "stabilize greenhouse gas concentrations in the atmosphere at a level that would prevent dangerous anthropogenic interference with the climate system." The framework sets nonbinding limits on GHG emissions for individual countries, with no enforcement mechanisms. An annual Conference of the Parties (COP) assesses progress in dealing with climate change, and these have occurred since 1995, with many producing specific international treaties (“agreements” or “protocols”) around GHG emissions. This history includes (a) the Kyoto protocol—a legally binding agreement that industrialized countries will reduce their emissions of GHGs, adopted in 1997 in Kyoto, Japan; (b) the Copenhagen Accord (2009), which recognized climate change as one of the greatest challenges of the present day and suggested actions should be taken to keep temperature increases to below 2°C; however, the document was not legally binding and does not contain any binding commitments for reducing CO_2_ emissions; (c) the Durban Platform (2011), which included a decision to adopt a legally binding treaty on climate change; the treaty terms were to be set out by 2015 and become effective in 2020 and for the first time, it included developing countries and the United States; and (d) the Paris Agreement (2015) was a climate change accord agreed by ~200 countries in December 2015 that came into force on November 4, 2016, committing world leaders to keeping global warming below 2°C and pursuing a tougher target of 1.5°C. Carbon emission curbs are not legally binding, but the framework of the accord, including a mechanism for periodically increasing pledges, is binding.

Since the FAR, the understanding of human influence on warming has improved through better earth observations and earth system modeling, with the AR5 stating that: (a) “Warming of the climate system is unequivocal, and since the 1950s, many of the observed changes are unprecedented over decades to millennia"^8^; (b) "Atmospheric concentrations of carbon dioxide, methane, and nitrous oxide have increased to levels unprecedented in at least the last 800 000 years"^8^; and (c) “Human influence on the climate system is clear…It is extremely likely (95%‐100% probability) that human influence was the dominant cause of global warming between 1951‐2010.”^8^ Special reports such as the recent SR15^9^ (global warming of 1.5°C) examined the difference in impacts between 1.5 and 2°C warming, suggesting that limiting warming to 1.5°C “is possible within the laws of chemistry and physics but doing so would require unprecedented changes” with “‘rapid and far‐reaching’ transitions in land, energy, industry, buildings, transport, and cities.” A 1.5°C limit would require global net anthropogenic CO_2_ emissions to decline by about 45% from 2010 levels by 2030, reaching net zero around 2050. To limiting global warming to below 2°C, CO_2_ emissions will need to decline by about 20% by 2030 and reach net zero around 2075.

We are already living in a 1°C warmer world. The SR15^9^ states “we are already seeing the consequences of 1°C of global warming through more extreme weather, rising sea levels and diminishing Arctic sea ice, among other changes … By 2100, global sea level rise would be 10 cm lower with global warming of 1.5°C compared with 2°C,”; and moreover, “Global warming is likely to reach 1.5°C between 2030 and 2052 if it continues to increase at the current rate (high confidence).” If we continue on our current emission trajectory, then we are likely to reach 2.6‐4.8°C warming by 2100, with recent modeling results suggesting that the higher climate sensitivities could be more likely. In terms of personal emission targets, average personal emissions in 2010 were around 5 tons per person and to reach the 2°C target would require reduction to around 1.5 tons by 2050.^10^ This is very ambitious and of is of course based on an equal sharing of emissions across the global population. In reality, the rich, Westernized nations use much more carbon per person than the poorer nations.^10^ There is the potential for major reduction in personal carbon footprints in the Western world typically by reducing the major contributors of car travel, heating, electricity use, and flying.

## 3 CLIMATE CHANGE: HEALTH AND DISEASE

**Figure 1:**
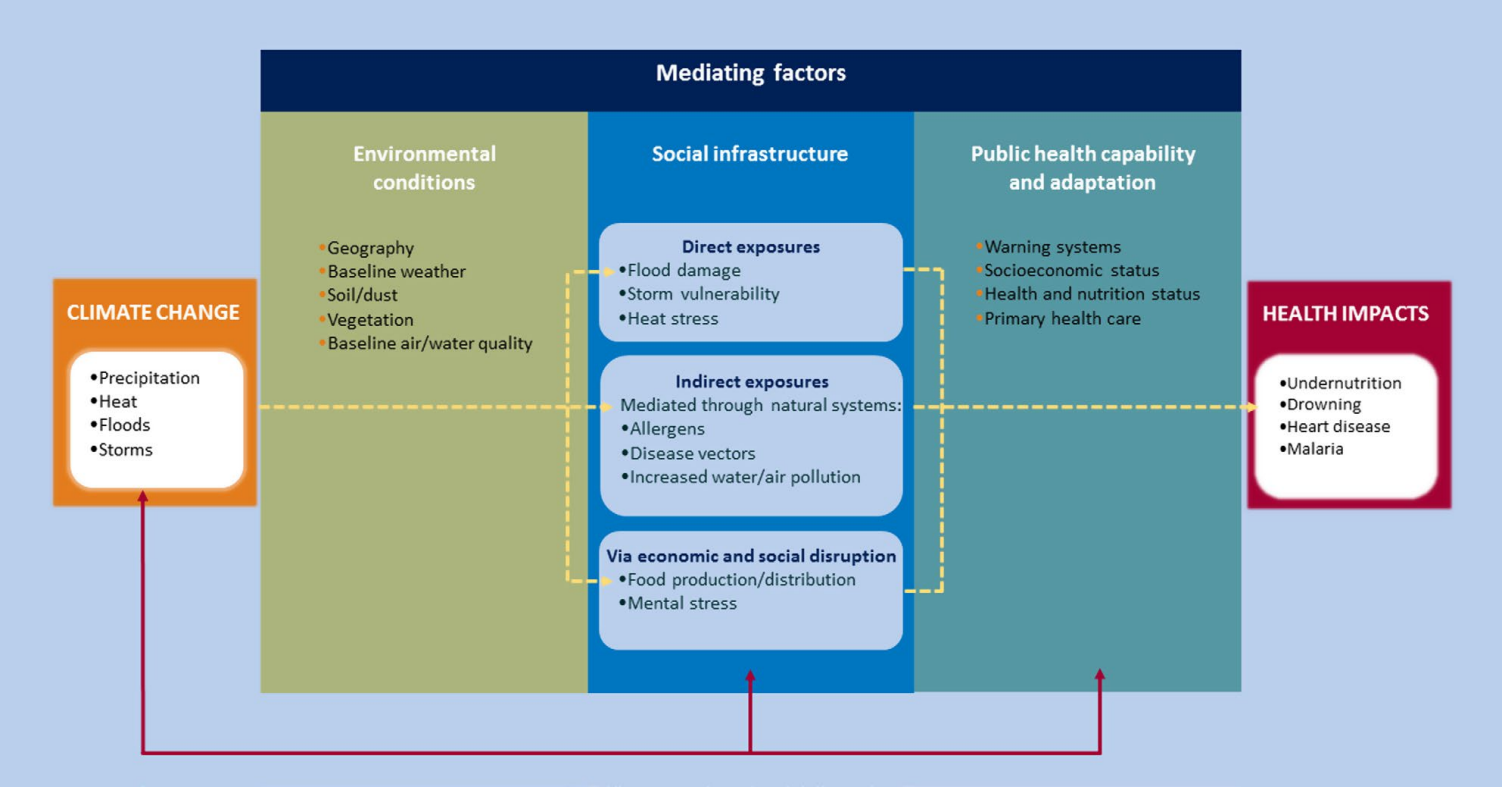
Pathways through which climate change may influence health.^1^

Existing variability in weather influences human health. For example, during clear summer days there is an increased occurrence of sunburn and Lyme disease, while during the winter, there is increased influence of influenza and falls. As climate change will influence weather, particularly weather extremes, then impacts on human health are likely. Hence, there has been an interest in the likely influence of climate change on human health for over 30 years, with a significant body of work now available on the health impacts of climate change.^11^, ^12^, ^13^, ^14^, ^15^ Climate change may lead to multiple health impacts acting through a variety of different pathways, as shown conceptually in Figure 1.

**Figure 2:**
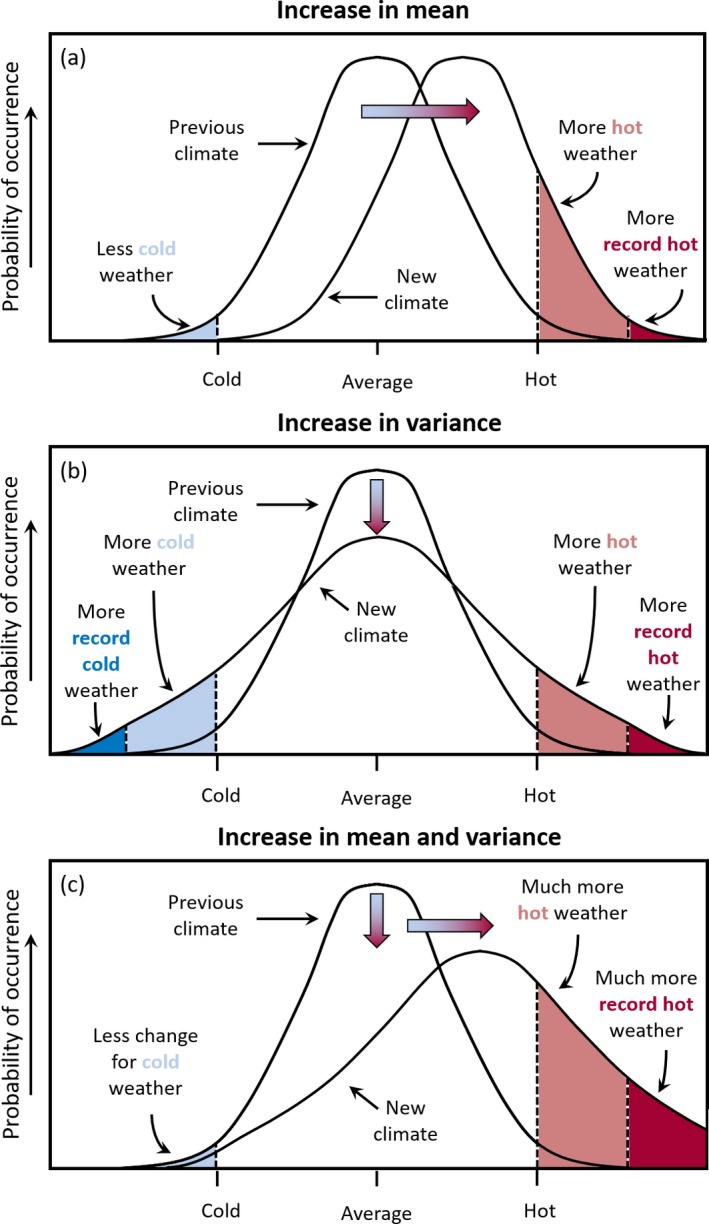
Schematic showing the effect on extreme temperatures when (A) the mean temperature increases, (B) the variance increases, and (C) when both the mean and variance increase for a normal distribution of temperature^1^

Climate change will affect mean weather, such as rainfall amounts and temperature. In addition to changing average conditions, it may also increase variability and extremes (eg, more intense rainfall events). The latter is probably of most consequence for human health. Although globally, interannual temperature has not increased in variability,^16^ there is evidence of regional changes in extremes,^17^,^18^ as both the mean climate and climate variability shift in response to anthropogenic forcings (Figure 2). These shifts in weather will be moderated by local environmental conditions and human adaptation, leading to changes in three broad categories of exposure that have the potential to influence health. The first are direct effects such as the influence of a heatwave upon mortality.^19^ The second are indirect exposures such as changing weather altering the growing season and distribution of plants, affecting pollen production, which in turn impacts on allergic disease^20^; other examples include changing weather patterns affecting mosquito breeding and life cycles, leading to changes in malaria and dengue fever (see below).^21^ Changes in temperature and humidity alter the geography of vector‐borne diseases, by shifting vector breeding sites^22^ and altering the transmission windows of vector‐borne diseases.^23^ In many cases, more data are needed to characterize the relationships between vector, pathogen, and climate.^24^,^25^ Third, climate change may act through economic and social disruption, for example, leading to mental health effects from extreme events such as flooding^26^ or influencing global crop yields leading to increased hunger.^27^ Flooding, whether coastal, fluvial, or pluvial, carries direct risks of drowning^28^ as well as a range of health risks in the aftermath of the event, including the spread of water‐borne diseases,^29^ and the spread of microbial contaminants in buildings.^30^ The risk of exacerbating existing medical conditions, through disrupted access to medicines and medical facilities, is also a concern.

However, changing exposure to extreme weather does not automatically lead to health impacts, and the gray box in Figure 1 highlights the important moderating influence that health care and wider public health capability can have in ameliorating potential impacts. Put simply, appropriate health care and public health input can reduce health impacts. It is important to recognize that these capabilities are dynamic over time. Furthermore, they can respond (ie, adapt) to new situations. One prominent example of such an adaptation occurred in response to the 2003 European heatwave during which over 70 000 additional deaths were recorded.^19^ City dwellers are at particular risk of heat stress given the urban heat island effect.^31^ Modeling suggests that in summer 2003, when an estimated 735 people died of causes relating to extreme heat in central Paris, anthropogenic climate change increased the risk of heat‐related mortality by approximately 70%,^32^ illustrating the scale of the issue. In response, many European countries developed early warning systems for heatwaves and national advice and guidelines on adaptation to reduce potential health impacts.^33^ Adapting public health capabilities will play an important role in responding to climate change.

The arrows in Figure 1 indicate feedback mechanisms between societal infrastructure, public health, and adaptation measures and climate change. Measures to reduce health impacts may also reduce GHG emissions. Examples include the health and environmental cobenefits of low carbon diets.^34^,^35^

## 4 CLIMATE MODELS: PROVIDING PROJECTIONS FOR PLANNING

Much of our understanding of likely impacts from climate change in the coming decades comes from climate models. Atmosphere‐Ocean General Circulation Models (AOGCMs)^36^ use the laws of physics to compute atmospheric, oceanic, and other environmental variables, and represent different processes and interactions in the climate system, at a series of evenly spaced “cells” or unit areas around the globe. Although important tools in our understanding, they are complex and require considerable computational resource to run. For this reason, the cells used by state‐of‐the‐art global climate models typically span a large area in order to reduce the number of calculations required: in turn, the outputs generated are coarse. While these outputs are useful for identifying global and regional trends, we need to adapt them for local use (eg, to assess the likely impacts of climate change in a particular coastal city or tropical community). This is accomplished using “downscaling” methods,^37^,^38^ which involve using either statistical approaches or a higher resolution model over a limited area to add greater detail.^39^,^40^ Unlike short‐term weather forecasts, which are dependent on the initial state of the atmosphere, longer‐term projections from climate models are largely dependent on trajectories of global development and on how fluctuating concentrations of GHGs impact the balance of sunlight absorbed by the Earth and energy radiated back into space. Thus, these longer‐term projections are subject to substantial uncertainty not related to the climate system. In the most recent model simulations, these trajectories are characterized by the representative concentration pathways (RCPs),^41^ a comprehensive dataset of changes to the Earth's energy balance under different possible futures. However, even when models are run with the same RCP, intermodel variability arises due to differences in how models represent processes, leading to different projected future climate states. For this reason, a multimodel ensemble is often used, in which uncertainty is considered to be represented by the spread of model projections.

The various choices and inferences made at each stage of the modeling process can therefore yield a substantial envelope of uncertainty in local impacts. Yet, despite these uncertainties, consistent findings with relevance for epilepsy emerge. For instance, models agree exceptionally well on estimates of heat stress metrics, which combine temperature and humidity.^42^ Furthermore, each generation of models represents an advance in our understanding of climate processes and a refinement of modeled processes. For example, for a number of extreme temperature indices, the spread among the most recent Coupled Model Intercomparison Project Phase 5 models (CMIP5; CMIP is a collaborative framework for supporting assessments of climate change and fostering model development, in which international modeling groups perform simulations based on preagreed scientific objectives, generating a database of model outputs with common standards) is less than the spread among the previous generation CMIP3 models, even though the project now contains more models.^43^

The burden of many of these changes will be felt by those least equipped to adapt. Models project increases in temperature variability in Amazonia, Southern Africa, the Sahel, India, and South‐East Asia over the coming decades.^44^ In India, models also indicate an intensification of heatwaves, with areas that do not currently experience heatwaves becoming severely affected this century.^45^ Thus, models can help us understand where we can expect the effects of climate change to be most marked and what those effects might be, in turn helping us plan for the likely challenges that will need to be addressed for health care in general, and epilepsy in particular.

## 5 IMPACTS OF CLIMATE CHANGE ON EPILEPSY

As the climate changes, we can expect there to be particular implications for epilepsy. These impacts might manifest at the level of altered risk of epilepsy per se, altered risk of seizures in established epilepsy, consequences for epilepsy treatments, and outcomes, with effects more likely to affect those not favored in the first place by geography and economics. We can expect inequalities in epilepsy health care to be further exacerbated.

Climate warming leads to spread of vectors for infections, which is already being reported, and more can be anticipated. Climate‐driven hyperthermia, fevers, food and water stress, and starvation may further compound other difficulties as well as being directly relevant themselves. Moreover, while humans are remarkably adaptable and have found niches in almost every terrestrial environment, climate change will superimpose unprecedented rates and types of change.

A number of vector‐borne infections can cause epilepsy or seizures in the acute phase. Chikungunya virus infection is associated with seizures as part of a multisystem acute illness in 10% of patients,^46^ while there are case reports of epilepsy as part of the chronic sequelae of neonatal chikungunya encephalitis.^47^ Epilepsia partialis continua has been reported with dengue encephalitis.^48^ Vertical Zika virus infection can lead to epilepsy even in the absence of microcephaly,^49^ and the epilepsy is typically drug‐resistant.^50^ If climate change leads to a spread of the vector carrying the infectious agent, then there will be an accompanying risk of spread of infection‐related epilepsy or acute seizures. Predictions are difficult, because the impact of climate change on vectors is multifactorial, and may be further compounded by multiplier effects as well as actions taking to mitigate its effects. It is already the case that the climate‐sensitive diseases affect the poorest populations the most, with higher prevalence and greater mortality, for several reasons.^51^ Outbreaks of vector‐borne infections can easily overwhelm healthcare systems, for example, as shown by recent outbreaks of Ebola and Zika viruses, and emergence of infections in naïve populations or those that have lost immunity can be even more dangerous. Many factors related to climate change can alter vector ranges, survival, biting behavior, pathogen infection capacity, and thus ultimately the risk of disease in humans. Changes in rainfall patterns, drought‐rain cycles, and flood magnitude and frequency can all have effects, while human strategies to combat climate change, such as reestablishment of wetlands and green spaces, can, perversely, promote vector spread. Accounting for these effects in model simulations is particularly challenging, but preliminary studies suggest, for example, that climate change will favor the spread of dengue fever and other arthropod‐borne infections given the temperature sensitivity of many factors favoring the spread of infection.^52^ Environmental, especially urban, effects of climate change might lead to the spread of cysticercosis,^53^ neurological involvement from which is a major cause of epilepsy globally. A study focusing on the risk of spread of vector‐borne disease in the UK resulting from climate change suggests that a 1°C rise in temperature would permit Chikungunya virus transmission for 1‐3 months of the year across most of southeast England by 2071‐2100, with changes in transmission ranges also found for a range of other arthropods.^52^

Malaria is caused by five species of Plasmodium transmitted by Anopheles mosquitoes. In malaria‐endemic areas, up to 90% of the population may have parasites detectable in their blood. *Plasmodium falciparum* and *Plasmodium vivax* are associated with seizures and epilepsy,^54^ particularly seizures during acute infections, and development of epilepsy has been documented following severe falciparum malaria. In addition, there is evidence that asymptomatic malaria parasitemia can precipitate seizures in those with epilepsy.^55^ There are many models of the effect of climate change on the transmission of malaria, with the most significant and consistent effects predicted to occur in the highlands in Africa and parts of South America and South‐East Asia.^56^ In other regions, the effect of climate change on malaria transmission may be minimal due to improved control measures and socioeconomic factors, although the present decline in malaria transmission across the world depends on continued support for the current control measures. However, significant uncertainties exist in these models, particularly in the areas that experience malaria epidemics. Furthermore, most of these models examined the effect of climate change on mosquitoes that carry *P falciparum*, but it is the Anopheles species that transmit *P vivax* (particularly *Anopheles atroparvus*) that are most likely to increase seizures and epilepsy in Europe.^57^ For example, in one model, a medium‐high scenario of climate change predicts that the southern half of Great Britain will be climatically suitable for *P vivax* malaria transmission by 2030 and by 2080 even southern Scotland will be climatically suitable for 2 months of the year.^58^

Phenomena other than infections are also worsening with climate change. As conflicts arise, we can expect more head injuries as another cause of epilepsy. A study from a highly polluted city in China suggests that transient increases in air pollutants such as nitrogen dioxide and sulfur dioxide are associated with increased hospital visits for epilepsy, supporting findings from another study from Chile that reported an increased risk of hospitalization for epilepsy with increases in air pollutants.^59^ Another study identified a tentative link between higher relative air humidity and an increased risk of admission for an epileptic seizure (though higher ambient temperature was associated with a lower risk).^60^ These are preliminary studies that suggest the relationships, if correct, are complex with a need for more data. But, there is certainly a prevailing view that the outlook strongly favors strategies to forestall climate change rather than trying to deal with the consequences on epilepsy and seizures of the complicated environmental changes that will occur due to climate change.

Temperature can affect genes and proteins. Most genetic epilepsies are due to dysfunctional channels. As in much of epilepsy genetics, *SCN1A* is among the best studied in this regard. Some mutations in *SCN1A* that can cause epilepsy lead to defective folding of the encoded channel: Mutant channel insertion into a cell surface is temperature‐sensitive, for example, with increased insertion and current density at 30°C compared to 37°C.^61^ Mutations in *SCN1A* can cause the rare, severe developmental and epileptic encephalopathy Dravet syndrome. While the effect of such dynamic changes in the context of prolonged periods of higher ambient temperature in thermoregulating humans is difficult to predict, empirical and modeling data show direct effects of higher temperatures on sodium channel biophysical properties and neuronal dynamics.^62^ Elevated ambient temperatures due to hot baths increase seizure frequency in Dravet syndrome.^63^ Anecdotally, the UK Dravet syndrome patient support group reported that during the sustained atypical high ambient temperatures of the summer of 2018, affected children experienced more seizures and greater lethargy, in keeping with survey data from the Netherlands.^64^ We previously reported the death of a young patient with Dravet syndrome who had been seizure‐free, but had been walking outside on the hottest day in Melbourne (Australia) for years, with a peak of 46.5°C.^65^ There are other temperature‐sensitive epilepsies, though generally less well studied. An epilepsy‐related mutation in *SCN8A* was associated with temperature‐sensitive protein expression and function.^66^ Other genetic epilepsies with fever‐induced seizures include those due to certain mutations in the genes *GABRG2*, *CHD2, STX1B*: Temperature elevation can affect brain thermal regulation and epileptiform discharges and induce seizures in models carrying certain mutations in *GABRG2* 67 or *STX1B*.^68^ Moreover, seizures in many epilepsies, of all types, are more frequent with psychological stress. Climate change is projected to increase population stress levels as it will affect mean climate, such as rainfall amounts and temperature, but also weather variability and extremes, probably of most consequence for human health.

**Figure 3:**
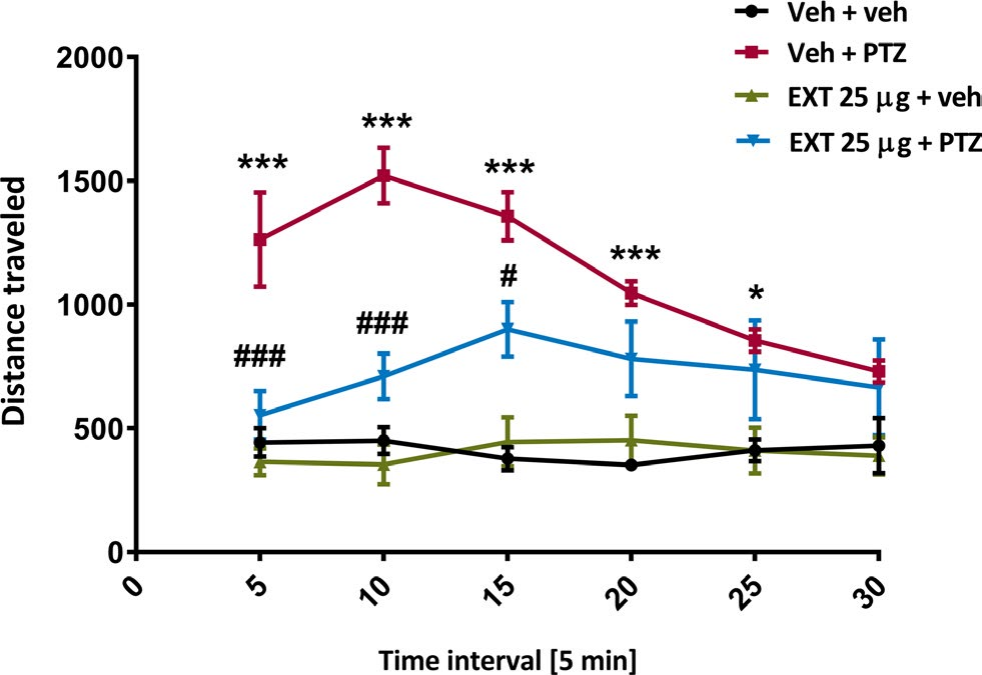
Example of a zebrafish behavioral assay showing anticonvulsant activity of a plant extract typically used for ethnomedicinal purposes to treat seizures in Cameroon. Zebrafish larvae were pretreated with the ethanolic plant extract (25 µg/mL) for 18 h before acute exposure to pentylenetetrazol (20 mmol/L). Pentylenetetrazol‐induced convulsive‐like behavior was measured 5 min after, using an automated video‐tracking device. Graph shows total distance traveled by larvae in millimeters per 5‐min interval during a 30‐min tracking session. Data were analyzed using specialized software to quantify total larval movements and subjected to two‐way with repeated‐measures ANOVA, followed by Bonferroni's *post hoc* test (n = 10‐12/group). ^***^ *P*<.001, ^*^ *P*<.05 (vs Veh + veh), ^###^ *P*<.001, ^#^ *P*<.05 (vs Veh + PTZ). EXT, extract; PTZ, pentylenetetrazol; Veh, vehicle. *Note:* Total distance traveled by PTZ‐treated larvae is lower at later time intervals as larvae reach the equivalent of status epilepticus at this stage and are less able to move due to loss of posture—therefore accounting for lack of statistical significance between extract‐treated larvae

There has been little research into the effects of climate change on treatments for epilepsy. Lorazepam, used in many parts of the world as an emergency treatment for seizures, is especially prone to degradation with higher mean kinetic temperature.^69^ There are few data on other antiepileptic drugs (AEDs), and the summary of product characteristics for several commonly used AEDs do not include specific temperature recommendations, while, for example, phenobarbital tablets should be stored below 25°C.^70^ Medication supply chains can be compromised even today: It is easy to see how disruption to infrastructure could threaten AED (and indeed any drug) supply, as indeed happened after an exemplar (but not climate change‐related) disruption in a highly organized, well‐developed economy in the Great East Japan earthquake of 2011.^71^ In addition, biodiversity loss is a feature of climate change: This may compromise sources, such as plant, microbial, and fungal species, from which the next antiepileptic drugs might emerge (see Figure 3).

Moreover, poverty is associated with reduced access to health care, with the majority of people in the world today with epilepsy having no access to any AEDs. Most climate change projections anticipate that poverty is likely to increase, with disproportionate effects for those already most vulnerable. Thus, we can anticipate that the negative influence of epilepsy on socioeconomic status and all‐cause risk of premature mortality will grow with climate change independent of any direct effect on epilepsy biology.

## 6 CAN EPILEPSY CONTRIBUTE TO CLIMATE CHANGE?

As clinicians working in epilepsy, we seek to offer holistic care. For example, we consider comorbidities because their successful treatment is not only likely to be important per se, but also because the control of comorbidities may improve seizure control.^72^ We should, therefore, examine also whether there are facets of epilepsy care that might themselves contribute to climate change and thus, following the discussion above, to worsening quality of life for people with epilepsy.

At an individual level as professionals, clinicians contribute to climate change mainly through their travel choices and needs, especially flights; other sources of importance include energy consumption (such as leaving devices on standby; office temperature settings, and the purchase of goods). In the UK, richer people are responsible for more carbon emissions than poorer people: the 10% most wealthy (likely to include, eg, most doctors) generated 10.3 tons of carbon emissions, while the 10% least wealthy (likely to include an overrepresentation of people with epilepsy) generated 3.25 tons.^73^ Air travel overall may account for a small fraction of human carbon emissions, but is growing rapidly, and its environmental damage is difficult to engineer away. As an example, in Table 1, we list the additional carbon emissions generated in 2018 due to professional travel by three senior epilepsy clinicians.

**Table 1:** Carbon emissions for work air travel in 2018 and comparison with per capita emissions across the world (world data from 2016)

	Tons of carbon dioxide
SMS, neurologist in the UK	2.4 (additional emissions due to work flights)
UK average (per capita)	5.9 (per annum)
DHL, neurologist in the United States	3.7 (additional emissions due to work flights)
US average (per capita)	17.0 (per annum)
IES, neurologist in Australia	16.4 (additional emissions due to work flights)
Australia average (per capita)	17.0 (per annum)
Average figures	
World average (per capita)	4.8 (per annum)
Africa (per capita)	1.1 (per annum)
Europe (per capita)	7.6 (per annum)

Note

DHL, Daniel H Lowenstein; IES, Ingrid E Scheffer; SMS, Sanjay M Sisodiya

## 7 WHAT CAN THOSE INVOLVED IN EPILEPSY DO?

Measures to address climate change may include attempts to reduce carbon emissions, and actions for mitigation and adaptation.^1^ For epilepsy professionals, there may be steps that be included in daily practice. Raising awareness of climate change, its impact for people with epilepsy and our contributions to climate change is a start—the very purpose of this article. Concurrently, more research is required to investigate into climate change and epilepsy, to better define the risks and develop adaptations and mitigations.

Reduction of carbon emissions (“decarbonizing”) could begin with understanding the sources of carbon emissions at the individual level and monitoring of individual carbon contributions, for both professionals and people with epilepsy. Personal and SME carbon emissions calculators are available online (examples include: https://www.carbonfootprint.com/calculator.aspx; https://footprint.wwf.org.uk/#/; https://www.carbontrust.com/resources/tools/carbon-footprint-calculator/). Among the key contributors for most people as professionals will be travel, consumption of food and goods, IT equipment, hotels, and restaurants, but this is not an exhaustive list and we invite other suggestions.

Travel is likely to be a major contributor for many professionals and patients. Flying to conferences and meetings is likely to be an important component of contributions to emissions at the personal professional level. Flying in classes other than economy increases emissions significantly, for several reasons (eg, see http://blogs.worldbank.org/developmenttalk/blog-carbon-footprint-world-bank-group-staff-air-travel). Various websites, including some for academic travel in particular, can help people calculate, reduce, or mitigate emissions (eg, www.flyingless.org and https://thepointsguy.com/guide/a-guide-to-airline-carbonoffset-programs/). This is a difficult area, as clinical and scientific advances and stronger collaborations do emerge from face‐to‐face meetings, and teleconferencing still lacks both the personal touch and infallible reliability. But perhaps not every meeting is essential,^65^ and to achieve the carbon emission reductions necessary to stabilize the climate will require us to modify some current practices and find new ways to achieve the same outcomes. Targeted distribution of mitigation measures (eg, carbon offsetting) for conference attendance to those nations most at risk from climate change may suit some conference attendees. Higher levels of sustainability at conferences should be considered: hotel usage, maintenance of ambient temperatures in conference halls and hotels (and personal offices at work), and food and drink provision at meetings are all areas to be considered. Many organizations will consider these difficult changes to make for meetings that often represent sources of income, but as with many aspects of climate change, there is a bigger picture to consider. Climate scientists themselves have considered and addressed the issue of conference travel (https://noflyclimatesci.org/), even with a model for a “nearly carbon‐neutral conference.”^74^ More flying does not necessarily equate with greater academic success.^75^

Car journeys represent another area in which professionals and patients might both contribute, and actions such as telehealth initiatives may be possible in some settings and jurisdictions, though information technology infrastructure, privacy, and regulatory issues may present difficulties. Professionals can form powerful lobby groups and bring these issues to the attention of their organizations. We must not be nihilistic. Actions at governmental, institutional, and personal levels are required and possible. For example, the UK Health Alliance on Climate Change (http://www.ukhealthalliance.org/) advocates “for responses to climate change that protect and promote public health.” Online tools are available for further ideas and implementation of adaptation measures (eg, https://www.ukcip.org.uk/). Adaptations will be necessary and will need to be shaped by outcomes from research (ideally shared across diseases), such as improved awareness and management of changes in vector ranges or medicine manufacture and distribution practice.

Nondomestic contributions to climate change exceed those of the residential sector. Companies and institutions around the world are beginning to take action: The behavior of employees, including epilepsy professionals, may be overlooked in this process, but can be central to the initiation, promotion, and success of programs designed to address climate change. Asking employees to switch their computers off at the end of the day, for example, does not mean people will do that. Professionals can, however, choose to take the lead. Evidence shows that people can be motivated as much by “cobenefits” (such as economic and scientific advancement; or fostering a more moral and caring community) as they are by concern about climate change, whether they are convinced about the occurrence of climate change or not.^76^

Adaptations might include, for example, increased vigilance and preemptive changes in behavior against local extreme heat events for people with epilepsy due to heat‐sensitive mutation‐driven genetic epilepsies, informed by local (downscaled) modeling of future climate trends, or improved short‐term weather forecasting. As precision medicine initiatives become more popular, one could envisage inclusion of climate change parameters in individualized epilepsy management schemes.

We summarize some suggestions for actions in Table 2.

**Table 2:** Actions to consider regarding epilepsy and climate change

Type of action	Example
Research	
Increase information available on impact on climate change on epilepsy and vice versa	Apply for research funding; join initiatives addressing these issues
Collect data from people with epilepsy, own clinical practice	Establish contact with local and national patient and carer support organizations; gather data systematically
Evaluate potential local changes in climate	Establish links with local climate scientists, consider local and regional factors (eg, urban vs rural)
Report on observations	Changing patterns of seizures and comorbidities reported by people with epilepsy; use of social media and big data methods
Gather data on mechanistic and personalized medicine domains	Included temperature and humidity parameters in experimental models; quantification of stress and consequences in clinical trials; stability of medications
Steps available now	
Self‐education and information	Join local, national, international initiatives for individuals and professionals; calculate own carbon emissions at personal and professional levels
Develop practice approaches that are more sustainable	Consider changes with cobenefits to raise chances of engagement and success
Take specific measures	Consider travel options; use freely available tools; explore and engage with local health service sustainability efforts; lobby institutions, healthcare organizations, journals and conference organizers
For organizations	Consider measures that facilitate sustainable behavior—for example, earlier setting up of conferences to enable planning of more sustainable travel options
Support people with epilepsy	Provide information and advice—for example, when heatwaves are expected; promote telehealth where possible
Reduce emissions	Switch off devices when not needed; promote sustainable practices—for example, enable colleagues to attend meetings remotely, record talks for dissemination
Promote sustainability for the next generation	Facilitate remote supervision and mentoring; reduce dependence of academic progress upon less sustainable practices

## 8 CONCLUSIONS

Clinicians have always considered prevention to be better than cure. Minimizing the progress of climate change and mitigating emissions now will be cheaper than taking actions later when the situation has already gotten worse, although there is already a need for some adaptation due to warming from historical emissions. The challenge is huge, but it is incumbent upon us to do what we can: This is intrinsic to our commitment and duty to patient care and can be seen as part of the aim of achieving global equality of health care in epilepsy. People have managed huge challenges before—the reduction in drink driving rates, the reduced production of aerosols, and changes in behavior after the HIV epidemic. With climate change, the challenge is bigger, more global, and more urgent, but everything counts. Schools, towns, local councils, and nations have declared states of “climate emergency” (eg, https://www.bbc.co.uk/news/uk-politics-48126677), with a prominent role played by schoolchildren. Should and can a profession take action beyond virtue signaling? And if so, what additional evidence do we need to do that? Or in fact can we not afford to wait? We acknowledge the links between epilepsy and climate change we discuss are speculative, and more research is required. And, of course, anything we can do in our personal lives anyway can contribute to help counter one of the biggest challenges facing humanity and all life on this planet.

We should perhaps finish with the words of the next generation, specifically Greta Thunberg (https://en.wikipedia.org/wiki/Greta_Thunberg), aged 16, speaking to members of the European Parliament and European Union officials in Strasbourg on April 16, 2019, after the disastrous fire at Notre Dame cathedral: “It is still not too late to act. It will take a far‐reaching vision, it will take courage, it will take fierce, fierce determination to act now, to lay the foundations where we may not know all the details about how to shape the ceiling. In other words, it will take cathedral thinking. I ask you to please wake up and make changes required possible.”

## CONFLICTS OF INTEREST

The authors declare no conflicts of interest. We confirm that we have read the Journal's position on issues involved in ethical publication and affirm that this report is consistent with those guidelines.

## FUNDING INFORMATION

We are grateful to the Epilepsy Society for their support of this work and funding (SMS). This work was partly carried out at NIHR University College London Hospitals Biomedical Research Centre, which receives a proportion of funding from the UK Department of Health's NIHR Biomedical Research Centres funding scheme.
